# HADHB mediates 5-fluorouracil sensitivity in colorectal cancer

**DOI:** 10.1007/s12672-025-03503-1

**Published:** 2025-08-28

**Authors:** Xue Zhang, Hui Jin, Dan Li, Jiayin Liu, Jing Han

**Affiliations:** Department of Medical Oncology, The Fourth Hospital of Hebei Medical University, Shijiazhuang, China

**Keywords:** Colorectal cancer, 5-Fluorouracil sensitivity, HADHB, ROS

## Abstract

**Purpose:**

5-Fluorouracil (5FU) is a primary chemotherapy for colorectal cancer (CRC), but resistance reduces its effectiveness. HADHB, important in mitochondrial fatty acid β-oxidation, is linked to tumor metabolism changes in various cancers. Its potential influence on 5FU sensitivity in CRC remains unclear. This study aims to elucidate the role of HADHB in modulating 5FU sensitivity in CRC.

**Methods:**

Collect CRC tissue samples treated with 5FU and perform immunohistochemical staining to evaluate the relationship between HADHB expression and 5FU efficacy. We assessed the impact of HADHB on 5FU IC_50_ in CRC cells via CCK-8, confirmed HADHB-DUOX2 interaction through co-IP, and used fluorescence staining and flow cytometry to measure ROS levels. Metabolomics and transcriptomics were employed to investigate DUOX2-related metabolic pathways.

**Results:**

HADHB was significantly upregulated in 5FU-resistant CRC tissues compared to sensitive ones. HADHB knockdown in CRC cell lines improved 5FU sensitivity, increased apoptosis, and caused cell cycle arrest. We identified DUOX2 as a novel HADHB-interacting protein, with their protein levels showing strong positive correlation. Silencing either HADHB or DUOX2 can result in a decrease in ROS production, while DUOX2 overexpression reversed the ROS reduction caused by HADHB knockdown, thereby establishing a functional connection between these two elements in the regulation of ROS. This mechanism may play a crucial role in modulating the sensitivity to 5FU mediated by HADHB.

**Conclusion:**

HADHB overexpression is linked to 5FU resistance in CRC, indicating it as a potential therapeutic target, likely via the HADHB-DUOX2-ROS pathway.

**Supplementary Information:**

The online version contains supplementary material available at 10.1007/s12672-025-03503-1.

## Introduction

Colorectal cancer (CRC) is the third most common cancer and the second deadliest worldwide [1]. According to the American Cancer Society, the burden of CRC is rapidly shifting towards younger patients and more advanced metastatic cases [1]. Although immunotherapy has emerged as a prominent area of research in oncology, characterized by extensive investigation into treatment modalities and the immune microenvironment across diverse cancer types [2–4], its effectiveness in CRC remains constrained. Only about 5% of patients with microsatellite instability-high (MSI-H) or deficient mismatch repair (dMMR) subtypes exhibit sensitivity to immunotherapeutic approaches [5]. Consequently, chemotherapy and targeted therapy continue to predominate in the treatment of CRC, with chemotherapy retaining its status as the foundational treatment strategy [6]. 5FU is widely used in various stages of CRC treatment, yet its resistance significantly impacts the efficacy. A comprehensive investigation into the mechanisms of 5FU resistance is essential for improving CRC patients’ prognosis.

HADHB, an enzyme integral to fatty acid oxidation and a component of the mitochondrial trifunctional protein in conjunction with its α-subunit [7], primarily catalyzes the synthesis of long-chain and medium-chain acyl-CoA esters. These esters are crucial substrates for the mitochondrial β-oxidation pathway, a vital process in fatty acid metabolism [8]. However, the role of HADHB in modulating sensitivity to 5-fluorouracil (5FU) and its involvement in the generation of reactive oxygen species (ROS) has not been thoroughly elucidated in previous studies.

Our prior research has indicated that dual oxidases 2 (DUOX2) is integral to 5FU sensitivity [9]. However, the upstream and downstream regulatory mechanisms associated with DUOX2 remain inadequately characterized. DUOX2 is a NADPH oxidase that exclusively produces H₂O₂ under calcium-dependent conditions. As H₂O₂ is one of the predominant ROS in biological systems, DUOX2 serves as a key enzymatic ROS source. In gastrointestinal inflammatory diseases, DUOX2 not only plays a role in immune response mechanisms but also leads to substantial production of ROS. Continuous activation of ROS mediated by DUOX2 has been significantly associated with the onset of CRC [10]. Furthermore, studies have found a close correlation between ROS generation and the resistance of CRC cell lines to 5-FU [11]. However, the underlying mechanisms remain to be elucidated.

In this study, we identified DUOX2 as a downstream interacting protein of HADHB and elucidated their roles in the production of ROS in CRC cells. Our research demonstrated that the knockout of HADHB results in a decreased proportion of CRC cells in the G0/G1 phase, significantly elevates the apoptosis rate, and enhances the sensitivity of CRC cell lines to 5FU. These findings offer novel insights and a theoretical foundation for enhancing the sensitivity of CRC patients to 5FU. Furthermore, we have, for the first time, unveiled the potential critical role of DUOX2, as a downstream protein of HADHB, in lipid metabolism, providing a new perspective for a deeper understanding of the relationship between HADHB and DUOX2.

## Materials and methods

### Human CRC tissue samples

Formalin-fixed paraffin-embedded specimens were obtained from 72 CRC patients between 2009 and 2019 for construction of tissue microarray (TMA). All patients underwent surgical resection followed by postoperative adjuvant therapy with 5FU. The cohort was stratified into two groups based on the occurrence of recurrence or metastasis within 6-months post-therapy: 5FU-sensitive and 5FU-insensitive groups. Prior to TMA construction, all cases were pathologically confirmed as colorectal adenocarcinoma with ≥ 30% tumor cellularity based on H&E-stained section (4-µm thickness, stained with Harris hematoxylin for 5 min and eosin for 1 min). Complete clinicopathological data (AJCC 8th edition TNM staging, differentiation status, and survival outcomes) were collected. This study was approved by the Institutional Review Board of the Fourth Hospital of Hebei Medical University with waived informed consent, in compliance with the Declaration of Helsinki.

### Cell culture

The human CRC cell lines HCT116 and SW480 were purchased from the Type Culture Collection of the Chinese Academy of Sciences (Shanghai, China). All cell lines were authenticated using short tandem repeat (STR) profiling. HCT116 cells were cultured in RPMI 1640 medium (Thermo Fisher Scientific, Waltham, USA), while SW480 cells were maintained in Dulbecco’s modified Eagle’s medium (DMEM) (Thermo Fisher Scientific, Waltham, USA), supplemented with 10% fetal bovine serum, and 1% penicillin-streptomycin solution (100 U/mL penicillin and 100 µg/mL streptomycin). Cells were cultured at 37 °C in a humidified incubator with 5% CO_2_.

### CCK-8 assay

Cell survival was assessed using the CCK-8 assay (Dojindo, Beijing, China) in SW480 and HCT116 cells. After HADHB knockdown, cells were divided into: (1) scramble siRNA-NC, (2) siRNA-HADHB knockdown, and (3) blank control groups, seeded at 6 × 10^4^ cells/well in 96-well plates (Corning, NY, USA), and treated with ten concentrations of 5FU ranging from 0 to 80 µM. After 72 h, 100 µL of CCK-8 reagent was added, and cells were incubated for 1 h. The absorbance at 450 nm was measured using a microplate reader after conducting three independent replicates for each group. Concentration-effect curves were plotted, and half-maximal inhibitory concentration (IC_50_) values were calculated.

### qRT-PCR

Total RNA was isolated using the TRIzol Reagent (Thermo Fisher Scientific, Waltham, USA) and subsequently reverse-transcribed into complementary DNA (cDNA) with a uniform RNA concentration across all samples, employing the Reverse Transcription System (Promega, WI, USA). The synthesized cDNA was then analyzed via quantitative polymerase chain reaction (qPCR) using the 7500 Real-Time PCR System (Applied Biosystems, USA) as previously described [12]. The primers of DUOX2 sequences were: Forward primer: 5’-GCTTGGACCCATCATTCAC-3’; Reverse primer: 5’-GCCGCAACCTCATAACCT-3’. The primers of HADHB sequences were: Forward primer: 5’-ATAAGGAATGTTGTGGTGGTGGATGG-3’; Reverse primer: 5’-TGTGAGCAGGAGTCTTGTCAGAGAA-3’. The comparative Ct method (ΔΔCt) was used to analyze relative expression of genes. The fold change was evaluated as 2^−ΔΔCt^. Three technical replicates per sample were presented.

### Flow cytometric analysis of apoptosis and cell cycle

Apoptosis in SW480 and HCT116 cells was evaluated using the Annexin V-FITC/PI Apoptosis Detection Kit (Sigma-Aldrich, St. Louis, MO, USA), while cell cycle distribution was analyzed by PI staining. Briefly, cells from different groups were seeded in 6-well plates (5 × 10⁵ cells/well) and cultured for 72 h. For apoptosis detection, harvested cells were resuspended in 195 µL binding buffer, incubated with 5 µL Annexin V-FITC for 15 min at room temperature in the dark, followed by the addition of 10 µL PI and incubation on ice for 5 min. For cell cycle analysis, cells were fixed in 70% (v/v) ice-cold ethanol for 2 h, washed with PBS, and stained with PI solution (50 µg/mL) containing RNase A (50 µg/mL) at 37 °C for 30 min in the dark. Finally, both apoptotic rates and cell cycle distributions (G0/G1, S, and G2/M phases) were simultaneously determined using a flow cytometer.

### IHC staining

The IHC staining procedure was performed on 4-µm-thick TMA sections following a standardized protocol. After deparaffinization in xylene and rehydration through graded alcohols, antigen retrieval was conducted in citrate buffer (pH 6.0) using microwave heating (95 °C, 15 min). Sections were then incubated with 3% H_2_O_2_ to block endogenous peroxidase activity. Primary antibodies against HADHB (Sino Biological, Beijing, China) and DUOX2 (Bioss Antibodies, Beijing, China) were applied at a dilution of 1:200 overnight at 4 °C, followed by incubation with secondary antibody (ZSGB-BIO) for 30 min at 37 °C. 3,3’-diaminobenzidine (DAB) was used as the chromogen with hematoxylin counterstaining. Protein expression was evaluated using a semi-quantitative scoring system that considered both the percentage of positive cells (0: ≤5%; 1: 6–25%; 2: 26–50%; 3: 51–75%; 4: >75%) and staining intensity (0: negative; 1: weak; 2: moderate; 3: strong). The final IHC score, determining low (score < 4) or high (score ≥ 4) expression, was calculated by multiplying these two parameters. All slides were independently assessed by two independent pathologists.

### Co-immunoprecipitation (co-IP)

HCT116 cell lysates were divided into three groups: Input, IP, and IgG. The Input group received protein loading buffer, while 2 µg of anti-DUOX2 or anti-HADHB antibody was added to the IP group, and 2 µg of antibody to the IgG group. Incubation was performed overnight at 4 °C, followed by SDS-PAGE separation and silver staining. Notable differences were observed between the IP group and Input/IgG groups, with fewer silver-stained protein bands in the IP group. Presence of bait protein-sized bands was confirmed successful co-IP. For immunoprecipitation, lysates were incubated with specific antibodies and protein A/G agarose beads overnight at 4 °C. After six washes, the beads were mixed with loading buffer, boiled for 5 to 10 min, and analyzed by WB.

### Western blot (WB) analysis

Cells were lysed using RIPA buffer supplemented with protease inhibitor cocktail (Roche Diagnostics, Basel, Switzerland) on ice for 30 min. Protein concentrations were determined using bicinchoninic acid (BCA) assay (Thermo Fisher Scientific, Waltham, USA) according to manufacturer’s instructions. Equal amounts of protein (30 µg per lane) were separated by 10% SDS-PAGE (Bio-Rad, 4561033) and transferred to PVDF membranes (Millipore, IPVH00010) using a wet transfer system (Bio-Rad) at 100 V for 90 min. Membranes were blocked with 5% non-fat milk in TBST for 1 h at room temperature before incubation with primary antibodies overnight at 4 °C: anti-DUOX2 (Santa Cruz, California, United States, 1:1000), anti-HADHB (Sino Biological, Beijing, China, 1:2000), and anti-GAPDH (Proteintech, Wuhan, China, 1:5000) as loading control. After washing with TBST (3 × 10 min), membranes were incubated with HRP-conjugated secondary antibodies (ZSGB-BIO, PV-6001, 1:5000) for 1 h at room temperature. Protein bands were visualized using ECL substrate (Millipore, WBKLS0500) and imaged with the ChemiDoc MP Imaging system (Bio-Rad).

### Knockdown of DUOX2 and HADHB

*DUOX2* and *HADHB* were knocked down in HCT116 and SW480 cells with siRNAs (Invitrogen, Carlsbad, CA, USA). The target sequence of DUOX2 was as follows: si-DUOX2, 5’-GGAGGACAACAUAGUGGUUTTAACCACUAUGUUGUCCUCCTT-3’. The target sequence of HADHB was as follows: siRNA-194, 5’-CGUUAGCCAAACCCAAUAUAATT-3’; siRNA-340, 5’-CCUAAGGAAGUAGUUGAUUAUTT-3’; siRNA-643, 5’-CGACUGUCUUUAAUCUCUAAATT-3’. A scramble siRNA was used as a negative control. Cells were seeded in 6-well plates at 2 × 10⁵ cells/well 24 h prior to transfection, with siRNA-Lipofectamine complexes prepared in Opti-MEM and added to cells at 60%-70% confluence. Following 6-hour incubation, the medium was replaced with complete growth medium. Knockdown efficiency was rigorously validated at both mRNA (qRT-PCR) and protein levels (WB analysis).

### Overexpression of DUOX2

To overexpress *DUOX2* in HCT116 and SW480 cells, an expression construct was generated by subcloning PCR-amplified full-length human DUOX2 cDNA into an EX-E1601-M02 vector (GeneCopoeia, Rockville, Maryland, America) and an empty vector was used as the negative control. The overexpression efficiencies were evaluated by qRT-PCR and WB analysis.

### Cellular ROS assay

Cellular ROS production was measured using a DCFH-DA probe-based ROS Detection Kit (Solarbio, Beijing, China) and flow cytometry. DCFH-DA was prepared in serum-free medium at 10 µM, and cells were incubated with it for 20 min at 37 °C, followed by three washes in serum-free medium. ROS levels were detected using flow cytometry (Beckman Coulter) at 488 nm excitation. Fluorescence microscopy under low light conditions was used for observation, and Image J software was employed for quantifying and comparing cellular ROS proportions.

### Statistical analyses

Data analyses were performed using GraphPad Prism software, version 5 and SPSS software 21.0. Differences were considered significant when *P* < 0.05. (^*^P < 0.05, ^**^P < 0.01, ^***^P < 0.001). The results are presented as the mean ± standard deviation. Differences between groups were evaluated with two-tailed Student’s t-tests. Correlation analyses were analyzed using the chi-square test. Kendall’s tau-b analysis was used to analyze the correlation between DUOX2 and HADHB proteins.

### Statement

All methods were carried out in accordance with the Declaration of Helsinki and the approved guidelines and regulations of the Medical Ethics Committee of the Fourth Hospital of Hebei Medical University.

## Results

### HADHB is associated with 5FU sensitivity in CRC

**Fig. 1:**
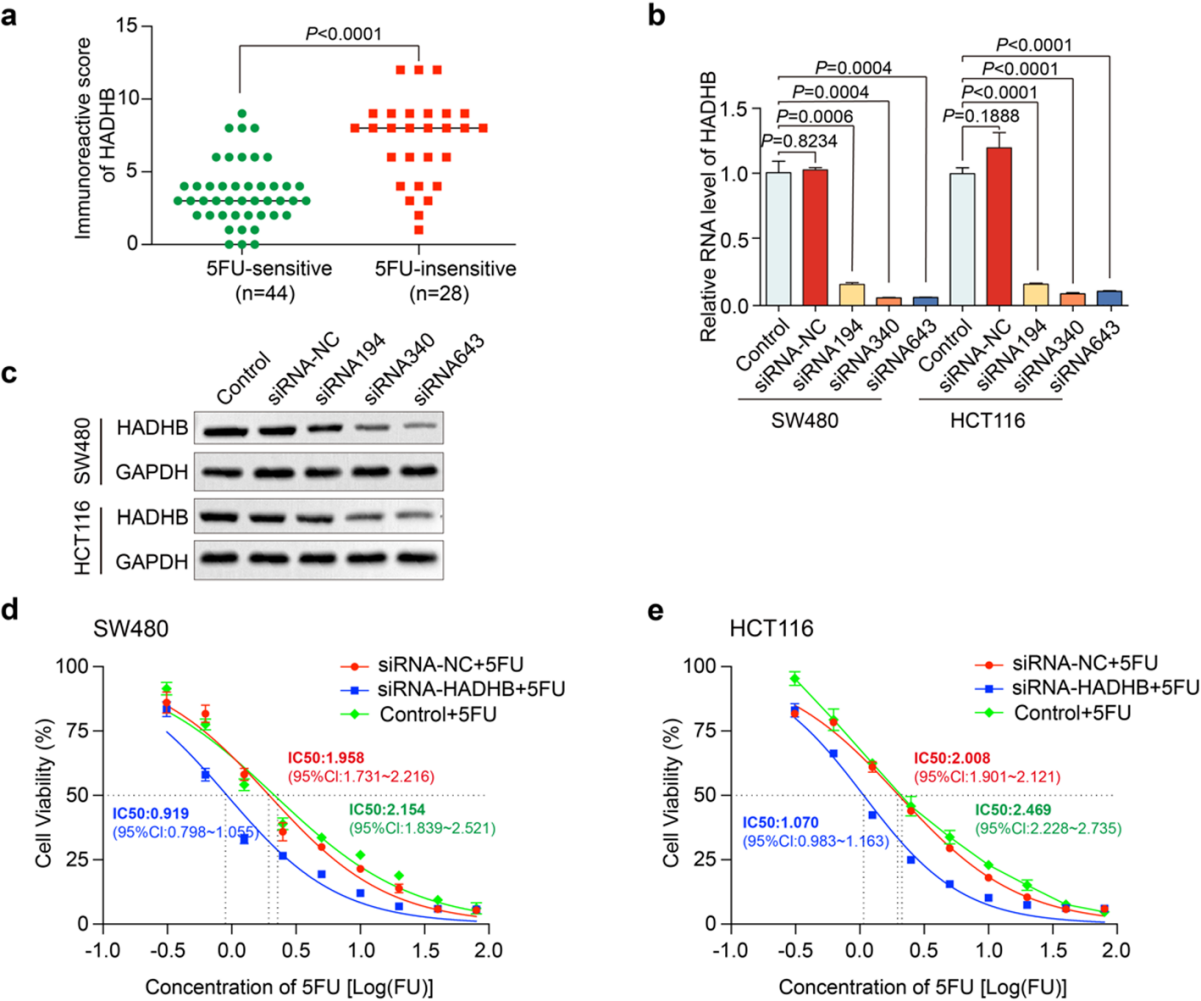
HADHB is associated with 5FU sensitivity in CRC. **a** Comparison of the IHC scores of HADHB protein in 5FU-sensitive group (*n* = 44) and a 5FU-insensitive group (*n* = 28) of CRC patients. **b, c** Three siRNAs were employed to achieve the knockdown of HADHB. The efficacy of this knockdown was subsequently validated through qRT-PCR and WB analyses. **d, e** Compared with the control group, HADHB knockdown significantly lowed the IC_50_ of 5FU both in SW480 and HCT116 cells

Postoperative cancer tissue samples were collected from 72 CRC patients and categorized into two cohorts based on their recurrence or metastasis status within 6 months post-adjuvant therapy with 5FU. The cohorts comprised a 5FU-sensitive group (*n* = 44) and a 5FU-insensitive group (*n* = 28). IHC analysis revealed a significantly elevated protein expression of HADHB in the cancer tissue specimens of the 5FU-insensitive group compared to the 5FU-sensitive group (Fig. 1a). To further investigate the role of HADHB in modulating 5FU sensitivity in CRC cells, siRNAs were designed to suppress HADHB gene expression. Subsequently, siRNA194, siRNA340, siRNA643, and a negative control (siRNA-NC) were transfected into HCT116 and SW480 cell lines. qRT-PCR and WB analysis demonstrated that siRNA340 and siRNA643 effectively reduced HADHB expression in both HCT116 and SW480 cells (Fig. 1b, c). To examine the impact of HADHB on the sensitivity of CRC cells to 5FU, we conducted transfections of SW480 and HCT116 cell lines using siRNA NC and siRNA643. A blank control group was also established for comparison. The CCK-8 assay showed a trend toward reduced IC_50_ values in the siRNA340 group compared to siRNA-NC. IC_50_ values of 5FU in the siRNA-mediated HADHB knockdown group were decreased to 46.9% and 53.3% compared to the control group in SW480 and HCT116 cell lines, respectively. These preliminary observations suggested a potential role of HADHB knockdown in modulating 5FU sensitivity that may require further validation (Fig. 1d, e).

### The impact of HADHB on apoptosis and cell cycle in CRC cells

**Fig. 2:**
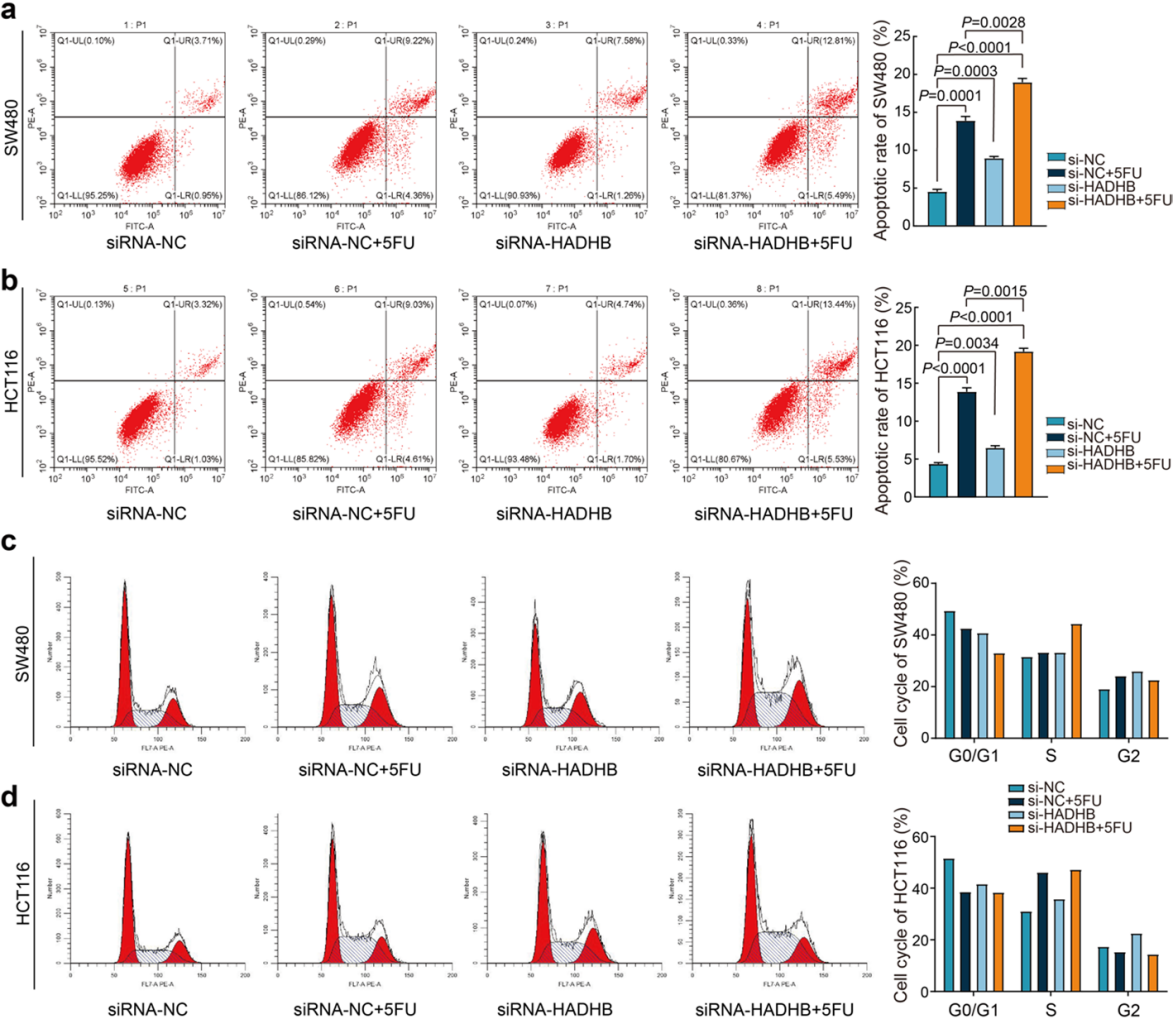
The impact of HADHB on apoptosis and cell cycle in CRC cells. **a, b** Flow cytometry was used to detect the effect of HADHB on the apoptosis rate of SW480 and HCT116 cells induced by 5FU. **c, d** Flow cytometric analysis of cell cycle arrest following HADHB knockdown and 5FU treatment

The flow cytometry analysis demonstrated a significant increase in the apoptosis rate of CRC cells following 5FU treatment. Furthermore, the knockdown of HADHB independently induced about a 2-fold increase in apoptosis. Notably, the combination of HADHB knockdown with 5FU treatment resulted in a significantly higher apoptosis rate than 5FU treatment alone (Fig. 2a, b). It is well-known that 5FU treatment induces S-phase arrest in CRC cells. In this study, we employed flow cytometry to assess the impact of HADHB knockdown on cell cycle phase distribution. The findings reveal that HADHB knockdown alone induced cell cycle arrest effects comparable to those observed with 5FU treatment in SW480 and HCT116 cells. Notably, the combination of HADHB knockdown and 5FU treatment led to a pronounced S-phase cell cycle arrest (Fig. 2c, d). Collectively, these results suggest that HADHB knockdown may potentiate the pro-apoptotic and cell cycle arrest effects of 5FU on CRC cells, indicating a potential therapeutic synergy.

### Interaction and correlation between HADHB and DUOX2

**Fig. 3:**
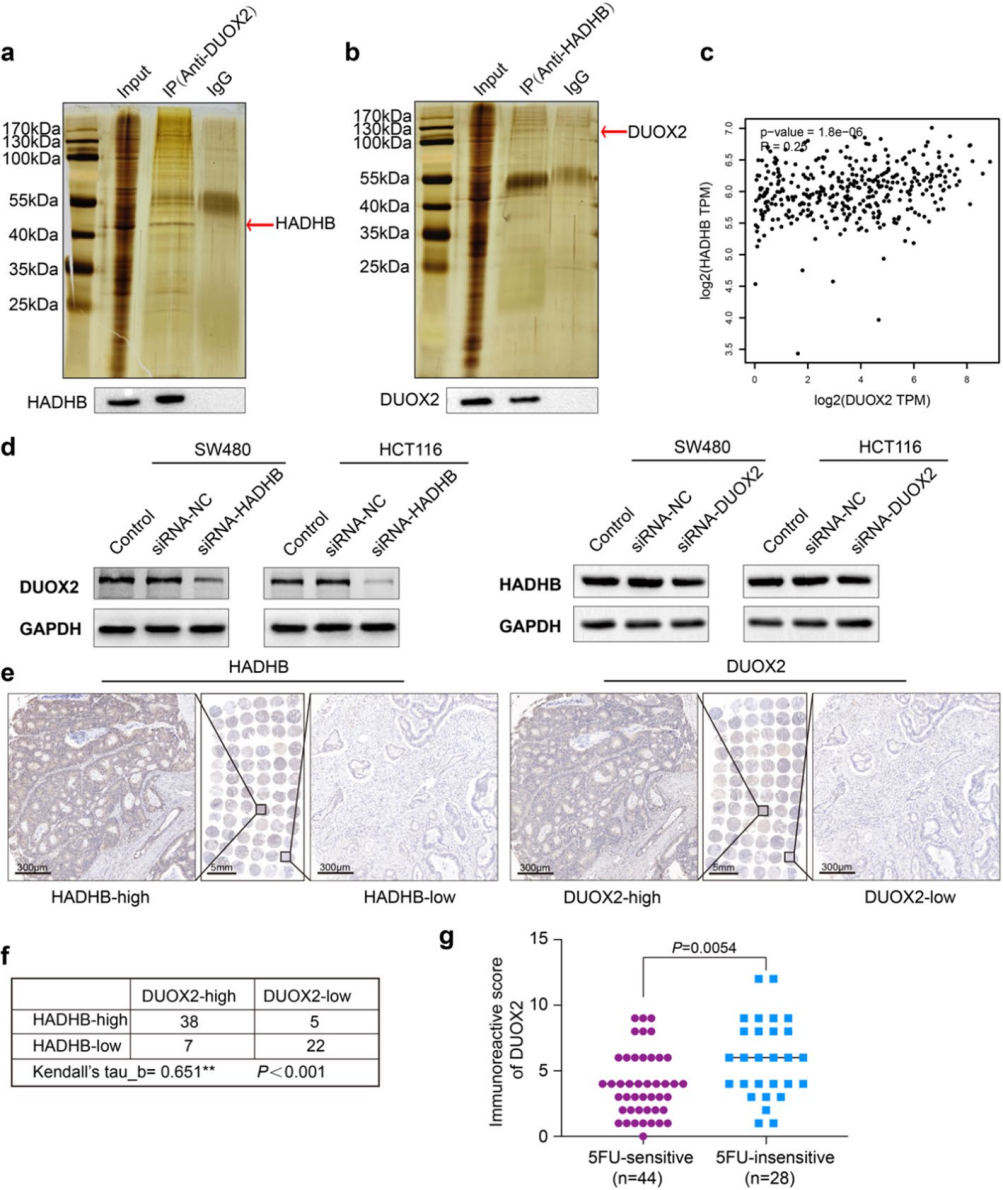
Interaction and Correlation between HADHB and DUOX2. **a, b** The interaction between DUOX2 and HADHB was demonstrated by means of co-IP. **c** The relation of *DUOX2* and *HADHB* in CRC tissues from the GEPIA database. **d** The DUOX2 and or HADHB protein expression after HADHB or DUOX2 knockdown in SW480 and HCT116 cells. **e, f** IHC staining of CRC TMA for HADHB and DUOX2. **g** Comparison of the IHC scores of DUOX2 protein in 5FU-sensitive group (*n* = 44) and a 5FU-insensitive group (*n* = 28) of CRC patients

In our prior investigations, IP-MS analysis suggested a potential interaction between the HADHB and DUOX2 proteins [12]. To further elucidate the molecular mechanism by which HADHB influences the sensitivity of CRC to 5FU, we employed co-IP analysis to validate the interaction between HADHB and DUOX2. The findings confirmed a direct interaction between these proteins (Fig. 3a, b). Subsequently, we examined the correlation between HADHB and DUOX2 expression levels, identifying a positive correlation between their mRNA levels in CRC tissues, as supported by data from the GEPIA database (Fig. 3c). Additionally, knockdown of HADHB in CRC cells resulted in a significant reduction in DUOX2 protein levels while DUOX2 knockdown did not substantially affect HADHB protein expression (Fig. 3d). This observation further supports the notion that HADHB acts as an upstream regulator of DUOX2, positively influencing its protein expression. Furthermore, IHC staining of CRC TMA for HADHB and DUOX2 also corroborated their positive correlation (Fig. 3e, f). We conducted an analysis of the correlation between DUOX2 protein expression and 5FU sensitivity in a cohort of 72 CRC patients. The findings indicated a significant upregulation of DUOX2 expression in the group exhibiting insensitivity to 5FU, which is consistent with HADHB (Fig. 3g).

### The effects of HADHB and DUOX2 on ROS generation in CRC cells

**Fig. 4:**
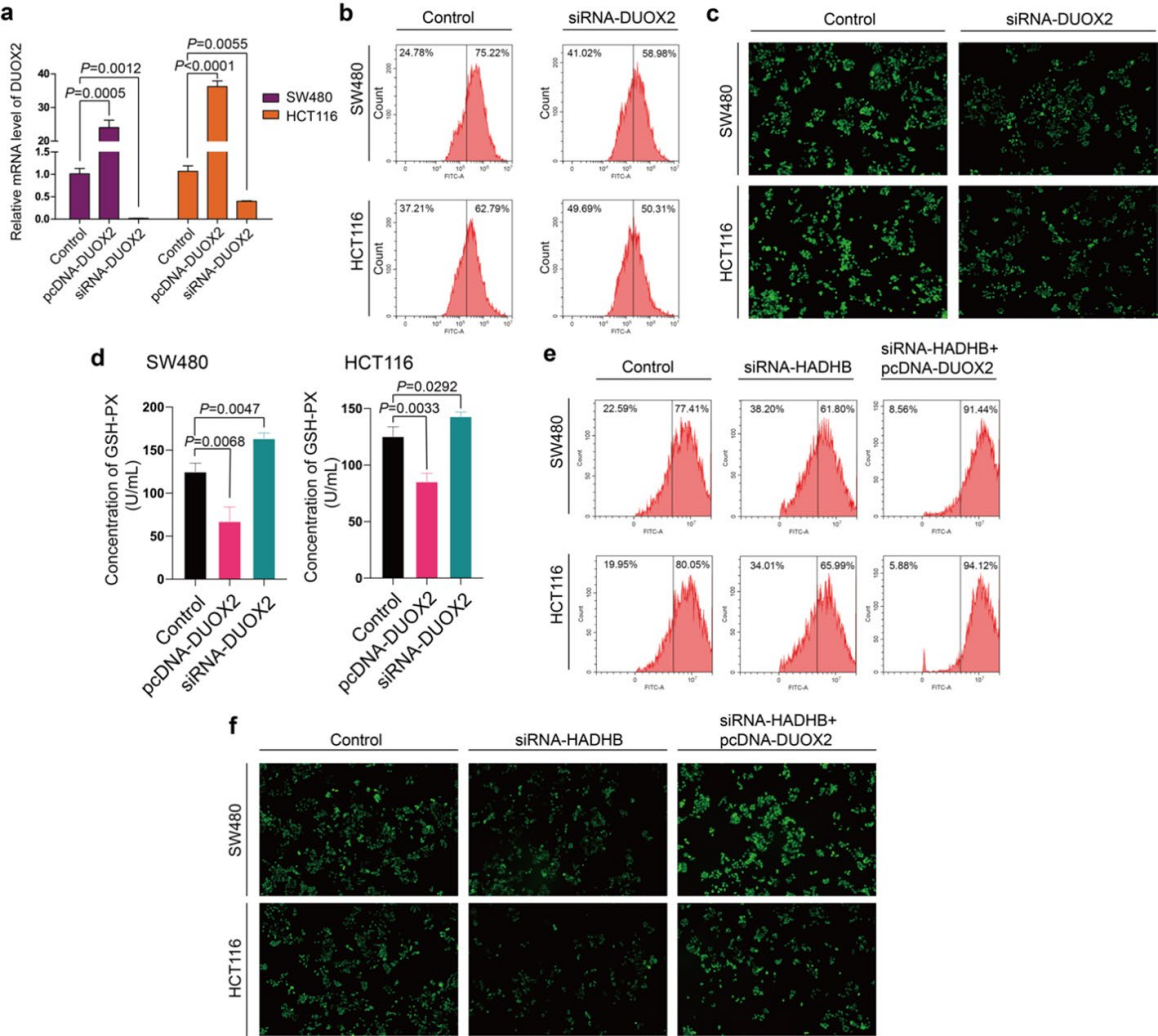
The effects of HADHB and DUOX2 on ROS generation in CRC cells. **a** The relative mRNA expression of DUOX2 after transfection with plasmids or siRNA in SW480 and HCT116 cells. **b** Evaluation of ROS levels using flow cytometry. **c** Fluorescence intensity shows changes in ROS content in CRC cells. **d** Biochemical analysis of changes in GSH-PX content after overexpression or knockdown of DUOX2. **e** Rescue assay. Flow cytometry shows that overexpression of DUOX2 can counteract the decrease in ROS levels caused by HADHB knockdown. **f** Rescue assay. The fluorescent probe is utilized to quantify intracellular ROS levels, with increased fluorescence signifying elevated ROS levels

As a member of the NADPH oxidase family, DUOX2 has been identified as being closely associated with the generation of ROS [13] and the chemoresistance of cancer cells [14]. In this study, we modulated DUOX2 expression in CRC cells through plasmid and siRNA transfection (Fig. 4a). Flow cytometry was employed to quantitatively evaluate ROS levels, revealing that DUOX2 knockdown led to a reduction in ROS levels (Fig. 4b). Fluorescence intensity was utilized as an indicator of ROS production, and a significant decrease in fluorescence intensity was observed in cells with DUOX2 knockdown compared to the control group (Fig. 4c). Glutathione peroxidase (GSH-PX), a critical antioxidant enzyme, plays a pivotal role in the detoxification of hydrogen peroxide (H_2_O_2_) and lipid hydroperoxides. Biochemical assays have demonstrated that the overexpression of DUOX2 in CRC cells results in a reduction of GSH-PX levels, whereas the knockout of DUOX2 leads to an elevation in GSH-PX content (Fig. 4d). These findings underscore the role of DUOX2 in ROS regulation. Given that DUOX2 is a downstream protein of HADHB, we further explored the effects of DUOX2 and HADHB on ROS production. It was observed that HADHB knockdown also reduced ROS levels, consistent with the effects of DUOX2 knockdown. Furthermore, DUOX2 overexpression was found to counteract the reduction in ROS levels caused by HADHB knockdown (Fig. 4e, f). These findings collectively demonstrate that HADHB regulates cellular ROS levels primarily through its downstream effector DUOX2 in CRC cells.

### Metabolomic insights into DUOX2’s role in CRC cells

**Fig. 5:**
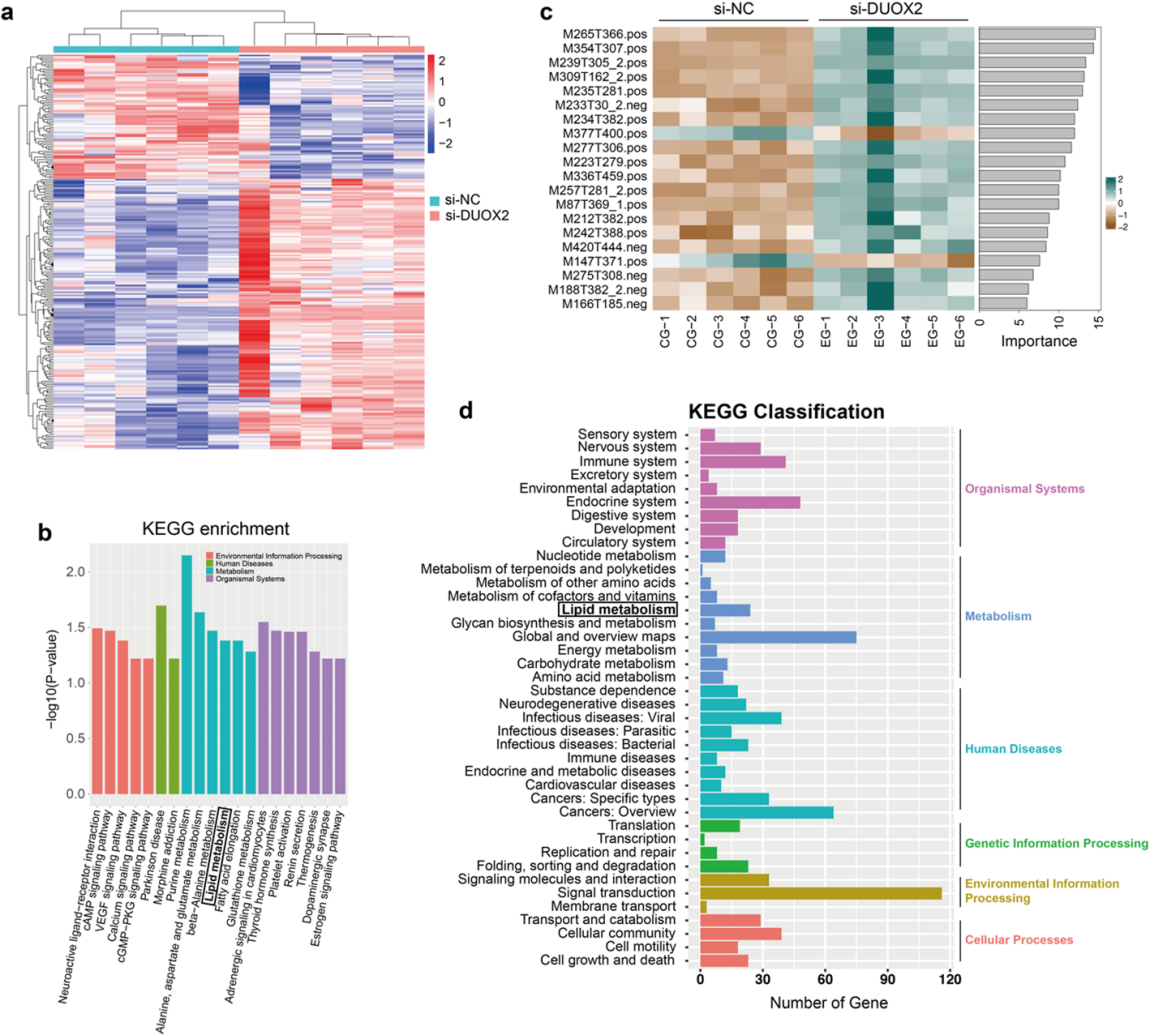
Metabolomic Insights into DUOX2’s Role in CRC Cells. **a** The heatmap analysis revealed significant disparities in metabolites between si-NC and si-DUOX2 SW480 cells. **b** The top 20 KEGG pathways with the most significant *P*-value. **c** The top 20 metabolites exhibiting the most significant differences between the si-NC and si-DUOX2 group. **d** Enrichment analysis of KEGG pathway of differentially expressed genes downstream of DUOX2 in CRC cells after knocking down DUOX2 by transcriptomics

Considering the crucial role of HADHB in fatty acid metabolism [15] and DUOX2 in ROS generation, we hypothesized that HADHB might promote ROS production through the regulation of fatty acid metabolism. To further elucidate the role of DUOX2 in the metabolism of CRC cells, we conducted an investigation into the metabolic differences between si-NC and si-DUOX2 groups using non-targeted metabolomics (Sup Table 1). The heatmap analysis revealed significant disparities in metabolites between the two groups (Fig. 5a). Comprehensive analyses were performed in both positive and negative ion modes to examine variations among all metabolites. Differential metabolites underwent KEGG pathway analysis, which confirmed their significant enrichment in pathways related to fatty acid elongation and degradation. The top 20 pathways with the most significant *P*-value enrichment were identified, including those associated with fatty acid metabolism (Fig. 5b). The top 20 metabolites exhibiting the most significant differences between the si-NC and si-DUOX2 groups are presented in Fig. 5c. Following the knockdown of DUOX2 in CRC cells, transcriptomics was performed. The differentially expressed genes downstream of DUOX2, as identified through Kyoto Encyclopedia of Genes and Genomes (KEGG) pathway analysis, also indicated that DUOX2 predominantly participates in lipid metabolism (Fig. 5d). Collectively, these findings underscore the potential critical involvement of HADHB, potentially mediated through DUOX2, in the lipid metabolism of CRC cells, thereby establishing a foundation for future mechanistic investigations.

## Discussion

Since its introduction in 1957, 5FU has been extensively employed in the treatment of various cancer types, particularly in CRC where 5FU and its prodrug derivatives have been consistently utilized as first-line therapeutic agents [16]. However, the clinical efficacy of 5FU in CRC is significantly hampered by the development of drug resistance. Therefore, unraveling strategies to enhance the sensitivity to 5FU treatment and to reverse the 5FU resistance remains a critical challenge in clinical practice. This necessitates a comprehensive understanding of the underlying mechanisms of 5FU resistance in CRC, and the exploration of potential synergistic approaches that could potentiate the efficacy of 5FU.

ROS are highly reactive oxygen-based molecules, primarily generated by enzymatic activity in organelles such as mitochondria, endoplasmic reticulum, and peroxisomes, encompassing both free radicals like superoxide anion($\:{\text{O}}_{2}^{-}$) and non-radicals like hydrogen peroxide (H_2_O_2_). ROS play a paradoxical role in cancer, acting as a double-edged sword. At the physiological level, ROS contribute to maintaining cellular homeostasis and are crucial for normal cell proliferation and differentiation [17]. In contrast, under pathological conditions, excessive ROS production may lead to various pathological changes, including cancer, inflammation, and organ damage [18]. Research indicates that a moderate increase in ROS levels can activate a multitude of proteins and signaling pathways through their oxidative capacity, thereby promoting tumor growth [19]. However, as ROS levels further escalate, cancer cells may undergo temporary or permanent proliferation arrest [20]. Elevated levels of ROS can lead to cancer cell death through various mechanisms, including autophagy, ferroptosis, Zheng et al. [21]. Theoretically, as primary mediators of oxidative stress, ROS appear to exert cytotoxic effects. Yet, in reality, most cancer cells are capable of establishing a dynamic equilibrium between high ROS levels and antioxidative responses, thus promoting cell proliferation while avoiding death [22]. Specifically, certain cancer cells exhibit persistent increases in ROS levels during chemotherapy, which might induce cell death due to heightened oxidative stress [23], but prolonged exposure to high levels of ROS can also enhance their resistance to chemotherapy [24]. It is worth noting that ROS-induced lipid peroxidation and DNA instability significantly promote cancer cell resistance to chemotherapy drugs [25]. Current research suggests that this resistance phenomenon is associated with multiple mechanisms of ROS production, including but not limited to the regulation of transcription factors [26], differentiation of cancer stem cells [27] and the tumor microenvironment [28]. Although the association of ROS with cancer has been extensively studied, the regulatory mechanisms of ROS production in CRC, as well as its potential impact on chemoresistance, still warrant comprehensive investigation.

However, the relationship between ROS production mediated by DUOX2 and tumor resistance remains elusive. Some studies suggest that DUOX2 may enhance cancer cell resistance to 5-FU by promoting ROS production, which triggers epithelial-mesenchymal transition in CRC cells [29]. Conversely, other research has found that an increase in ROS levels induced by DUOX2 can reshape the tumor microenvironment, thereby enhancing the sensitivity of glioblastoma cells to temozolomide [30]. These context-dependent effects suggest that the DUOX2-ROS may play a complex role in tumor resistance, with varying impacts depending on the type of tumor and treatment context.

Our research confirms that disrupting HADHB expression enhances the sensitivity of CRC cells to 5-FU, aligning with previous findings that reducing HADHB improves drug sensitivity in CRC cells [31]. Additionally, HADHB can regulate the expression of DUOX2 and promote the production of ROS in CRC cells in vitro, while overexpression of DUOX2 can reverse the ROS reduction caused by knocking down HADHB. Based on previous studies suggesting that ROS is involved in drug sensitivity, and our team’s previous research confirming the effect of DUOX2 on 5FU sensitivity, we speculate that the effect of HADHB on CRC’s 5FU sensitivity is achieved by targeting DUOX2 and regulating ROS generation. Numerous previous studies have well-documented 5FU’s impact on apoptosis induction and cell cycle arrest in CRC cells [32, 33]. We further observed that combined HADHB knockdown and 5-FU treatment significantly increased the efficiency of cell cycle arrest in CRC cells, suggesting that reduced ROS levels might help inhibit cancer cell proliferation. Prior research indicates that nM-range ROS concentrations are crucial for cell proliferation in response to growth factor stimuli [34], and slight increases in ROS can prompt cells to re-enter the cell cycle, thus effectively evading cell cycle arrest [35]. GSH-PX’s role in chemotherapy resistance is notable, as its expression level influences cancer cell sensitivity to chemotherapy. In CRC, GSH-PX expression is linked to oxidative stress changes post-chemotherapy, indicating its regulatory function in resistance [36]. The findings robustly support our research outcomes. This study proposes a potential mechanism through which HADHB may modulate ROS production via DUOX2, suggesting that targeting HADHB could be a viable strategy to alleviate 5FU resistance in CRC cells.

Lipid metabolism is a fundamental and complex biochemical process in the human body, influenced by various factors such as insulin, glucagon, dietary nutrition, and enzymatic activities, predominantly involving the metabolism of phospholipids and cholesterol. In this process, lipids are transformed into key components necessary for various biochemical reactions within the body [37]. Notably, HADHB plays a major role in the oxidative breakdown of long-chain fatty acids. This process, while providing energy for the cells, is usually accompanied by the substantial generation of ROS [38]. In cancer, the modulation of lipid metabolism plays a significant role in cell signaling, the composition of biological membranes, and other critical biological processes. Such metabolic regulation not only affects the proliferation and invasion capabilities of cancer cells but is also closely related to chemotherapy resistance [39]. Therefore, targeting the lipid metabolic pathways in cancer cells could emerge as an effective therapeutic strategy to enhance chemosensitivity [40].

To validate our hypothesis, we focused on analyzing the role of DUOX2, as the downstream protein of HADHB, in metabolic pathways. In this study, we found that DUOX2 may play a significant role in the elongation and degradation pathways of fatty acids, a discovery that has unexpectedly profound implications for our understanding of its functional significance. In the process of fatty acid metabolism, the elongation and degradation of fatty acids are two critical phases. Fatty acid elongation, a mechanism involving a continuous iterative process that incrementally adds two carbon atoms with each iteration, can physiologically promote the generation of nicotinamide adenine dinucleotide (NAD^+^) [41]. The activation of NAD^+^ may lead to excessive production of ROS in the mitochondria [42]. Concurrently, since the fatty acid elongation process depends on NADPH as an electron donor, and NADPH is a key factor in ROS elimination, the consumption of NADPH during fatty acid elongation could weaken the cell’s ability to clear ROS [43]. Additionally, fatty acid elongation significantly increases the quantity of unsaturated fatty acids, a process accompanied by substantial ROS generation [44]. These mechanisms collectively contribute to the increase in ROS production during fatty acid elongation. On the other hand, the degradation of fatty acids plays an essential role in maintaining energy supply for tumor growth, enabling cancer cells to rapidly access lipid reserves through the activation of lipophagy. The degradation of fatty acids primarily occurs in the mitochondrial electron transport chain, a process that generates a large amount of ROS. The activation of lipases in the fatty acid degradation process accelerates electron flux, further enhancing the formation of superoxide anions and hydrogen peroxide. Therefore, the degradation process of fatty acids is closely linked to ROS production, with both processes jointly influencing tumor growth and development [45].

Based on these findings, we have come to understand that there is a close connection between fatty acid metabolism and the levels of ROS production. Excessive ROS can induce genomic instability, alter gene expression, and participate in cancer-related signaling pathways. In CRC, these processes of fatty acid elongation and degradation are positively correlated with cancer cell proliferation and inversely associated with patient prognosis [46]. Therefore, we speculate that HADHB-DUOX2 might influence the biological characteristics of CRC by regulating fatty acid metabolism to increase ROS levels. This insight offers a new perspective for understanding the pathogenesis of CRC and may guide future therapeutic strategies.

In summary, our study identified HADHB as a key regulatory factor for 5FU sensitivity in CRC, possibly through the HADHB-DUOX2-ROS signaling pathway, in which lipid metabolism may also be involved. However, it is important to acknowledge several limitations in this study. Firstly, the precise molecular mechanisms underlying the interactions between HADHB and DUOX2 require further elucidation. Secondly, the physiological relevance of these interactions should be validated using appropriate in vivo models. Lastly, the intricate relationship between ROS signaling and lipid metabolic reprogramming necessitates more comprehensive investigation. These limitations highlight the critical need for further research to fully elucidate this regulatory network.

## Supplementary Information

Additional file 1.

## Data Availability

The datasets generated and analyzed in the present study are available in the GEO repository (GSE139918). The other data generated or analyzed in the present study are available from the corresponding author on reasonable request.
